# A yield-cost tradeoff governs *Escherichia coli’*s decision between fermentation and respiration in carbon-limited growth

**DOI:** 10.1038/s41540-019-0093-4

**Published:** 2019-05-01

**Authors:** Matteo Mori, Enzo Marinari, Andrea De Martino

**Affiliations:** 1 Department of Physics, University of California San Diego, 9500 Gilman Drive, La Jolla, CA 92093 USA; 2 Dipartimento di Fisica, Sapienza Università di Roma, Piazzale Aldo Moro 2, Rome, 00185 Italy; 3 INFN, Sezione di Roma 1, Piazzale Aldo Moro 2, Rome, 00185 Italy; 4 Soft & Living Matter Lab, Institute of Nanotechnology (CNR-NANOTEC), c/o Dipartimento di Fisica, Sapienza Università di Roma, Piazzale Aldo Moro 2, Rome, 00185 Italy; 5 Italian Institute for Genomic Medicine, via Nizza 52, Turin, 10126 Italy

**Keywords:** Bioenergetics, Biochemical networks, Systems analysis

## Abstract

Living cells react to changes in growth conditions by re-shaping their proteome. This accounts for different stress-response strategies, both specific (i.e., aimed at increasing the availability of stress-mitigating proteins) and systemic (such as large-scale changes in the use of metabolic pathways aimed at a more efficient exploitation of resources). Proteome re-allocation can, however, imply significant biosynthetic costs. Whether and how such costs impact the growth performance are largely open problems. Focusing on carbon-limited *E. coli* growth, we integrate genome-scale modeling and proteomic data to address these questions at quantitative level. After deriving a simple formula linking growth rate, carbon intake, and biosynthetic costs, we show that optimal growth results from the tradeoff between yield maximization and protein burden minimization. Empirical data confirm that *E. coli* growth is indeed close to Pareto-optimal over a broad range of growth rates. Moreover, we establish that, while most of the intaken carbon is diverted into biomass precursors, the efficiency of ATP synthesis is the key driver of the yield-cost tradeoff. These findings provide a quantitative perspective on carbon overflow, the origin of growth laws and the multidimensional optimality of *E. coli* metabolism.

## Energy metabolism balances carbon and protein costs

When growth conditions change, living cells adapt by tuning the allocation of resources (enzymes, ATP, etc.) to the different cellular tasks (in-taking nutrients, synthesizing ATP, etc.). This process links physiology, gene expression and metabolism, leading to major qualitative changes. Carbon overflow, i.e. the excretion of potentially useful carbon compounds that occurs in fast growing cells (including many types of tumors), is an example of this scenario. Our work addresses its origin by combining genome-scale mathematical modeling with empirical data analysis. Overflow in the bacterium *Escherichia coli* is found to stem from the trade-off between efficient nutrient usage (‘growth yield’) and the corresponding biosynthetic costs. This provides quantitative insight into the complex cellular economy underlying growth in different conditions.

## Introduction

The physiology of cell growth and its relation with gene expression and metabolism have been studied quantitatively for decades. Refining the picture developed since the 1950s,^1,2^ changes in growth conditions have been found to be accompanied by a re-organization of the cellular proteome, with resources being redistributed among different tasks (nutrient import, biosynthesis, etc.).^3–8^ For *E. coli*, this adaptation underlies significant adjustments in cellular energetics and metabolism, with the ‘acetate switch’^9–11^ being the most apparent manifestation of the interplay between metabolism and gene expression. Slowly growing *E. coli* cells tend to operate close to the theoretical limit of maximum growth yield (growth rate per unit of intaken carbon);^12–15^ at fast growth, instead, they display lower yields with the excretion of carbon equivalents (acetate). The transition between the two conditions is accompanied by large changes in gene expression.^6,11^ A similar scenario also occurs in other proliferating cell types, like yeast^16,17^ or cancer cells.^18–21^ Several phenomenological theories address the question of how metabolism and gene expression coordinate to optimize growth in bacteria,^4,11,22–27^ while mechanistic models of metabolism can describe changes in the usage of pathways at the crossover.^28–30^ Here we combine genome-scale modeling with experimental data analysis to obtain a direct quantitative characterization of the yield-cost tradeoff in *E. coli*.

We first show that, under carbon limitation, the growth rate can be explicitly decomposed into inter-dependent contributions from the growth yield and from growth-associated protein costs. Maximum growth then implies a tradeoff between the growth yield and the proteome fraction allocated to metabolic enzymes. At fast (slow) growth, the latter (former) is optimized, and the objective function changes smoothly as carbon availability is limited and one passes from one regime to the other. We quantitatively evaluated this scenario in a genome-scale model of *E. coli*’s metabolism. In this framework, maximum growth leads to a Pareto front in the yield-cost landscape. Remarkably, an analysis of proteomic data for carbon-limited cells^6^ validates this picture. To get insight into which metabolic variable drives the crossover, we further quantified the partitioning of carbon into biomass- and energy-producing pathways, both of which contribute to growth. While the relative amount of carbon converted into biomass precursors significantly exceeds that going into energy (i.e., ATP), the latter accounts for almost all of the observed change in growth yield at the crossover, implicating the ATP yield as the key biological driver of the acetate switch. The behavior of the corresponding proteome mass fractions extrapolated from data confirms this conclusion.

These results provide a quantitative characterization of the multidimensional optimality of *E. coli*^13,31^ that directly addresses the crosstalk between growth and gene expression by connecting metabolic flux analysis to proteomic data.

## Results

### Increasing the growth yield as a basic response to carbon shortage

Bacterial growth is known to be hindered by the synthesis of inefficient proteins.^3^ Because of this, modulating the proteome composition is a major fitness strategy for exponentially growing bacteria. In *E. coli*, for instance, a substantial reshaping of the protein repertoire takes place upon carbon limitation, with ribosome-affiliated proteins and biosynthetic enzymes taking up a smaller fraction of the proteome as the growth rate *μ* decreases, while carbon-import and motility proteins are upregulated.^3,5,6,8^ Similar changes are observed in cells subject to other modes of limitation.^6^ Such modifications indicate major shifts in growth strategy in response to stress.

For a minimal phenomenological description of this fact, let *J*_*C*_ denote the cellular carbon intake flux (in mmol/g_DW_h), which we assume to be subject to limitation (see Supplementary Text, Table S1 for a list of symbols used in this work). The carbon influx *J*_*C*_ is related to the growth rate *μ* via the specific intake flux *q* (i.e., *J*_*C*_ = *q* ⋅ *μ*), representing the amount of in-taken carbon needed to generate a unit of biomass (in units of mmol per gram of dry weight). *q* depends not only on the specific nutrient source, but more in general on the growth conditions (e.g., oxygen availability, osmolarity, pH). The inverse of the specific intake flux is proportional to the growth yield, which is usually expressed in terms of grams of dry weight per gram of intaken carbon; the specific carbon intake *q* is, however, more convenient for our purposes. For fixed specific intake *q*, as carbon is limited and the stress level increases, *J*_*C*_ decreases and *μ* is proportionally reduced. In turn, the proteome share *ϕ*_*C*_ allocated to the carbon import system will also be affected, as more proteins devoted to importing carbon will generically be needed to sustain a prescribed rate *J*_*C*_ under limitation. The cost associated to this macromolecular burden can be modeled via a coefficient *w*_*C*_ > 0 constraining the catabolic proteins and the carbon intake, *ϕ*_*C*_ = *w*_*C*_ ⋅ *J*_*C*_, and serving as a proxy for the level of stress to which the cell is subject. Putting things together, the growth rate *μ* can be expressed as

1 $$\mu = \frac{{\phi _C}}{{q \cdot w_C}}.$$

**Fig. 1:**
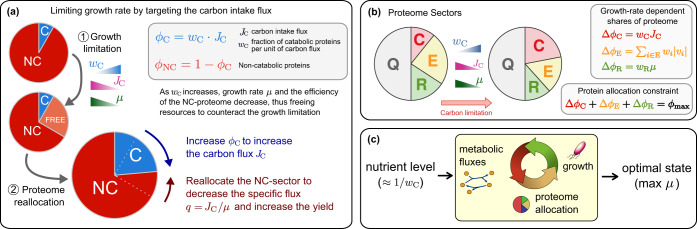
Schematic view of the proteome allocation problem. **a** Effect of growth limitation via carbon starvation on proteome composition. An increase in the cost of the catabolic (C) sector reduces the growth rate. Part of the non-catabolic (NC) sector, no longer required at slower growth, is freed and can be reallocated. The decrease in growth can be counteracted by expanding the C-sector (i.e., by increasing *ϕ*_*C*_) and/or by allocating part of the NC-sector to re-organize metabolism so as to decrease the specific carbon intake *q* = *J*_*C*_/*μ*. **b** Proteome sectors considered for *E. coli*, namely ribosomal (R), enzymatic (E), carbon-import (C), and housekeeping core (Q). Following ref. ^30^ we assume that all sectors but the core have condition-dependent parts denoted by Δ*ϕ*_*j*_. By normalization, their sums are constrained as in Eq. (3). **c** A change in the nutrient level affects the cellular metabolism and gene expression. In Constrained Allocation FBA,^30^ the interplay between metabolic fluxes, protein levels, and growth rate leads to optimal phenotypes with maximum growth rate

Eq. (1) suggests that cells can counteract an increase of *w*_*C*_ (i.e., higher stress associated to carbon limitation) in two ways (Fig. 1a). The first is by increasing the protein fraction associated to carbon transport, *ϕ*_*C*_: indeed, *E. coli* expresses a whole array of catabolic systems in response to carbon shortage.^5,6^ The second is by decreasing the specific intake rate *q*, i.e. increasing the growth yield. The switch from fermentative to respiratory metabolism upon carbon limitation is an example of this type of response,^9–11^ with the specific intake *q* reaching a minimum when acetate excretion flux is negligible. *E. coli* adopts both strategies under carbon limitation;^6^ the key difference between them is that, while the former only involves a specific set of processes (i.e., those involved in carbon uptake), the metabolic rewiring needed to decrease the specific intake *q* implies the coordination of expression levels across multiple metabolic pathways, even not directly subject to limitation.

### The role of protein costs

Any increase in *ϕ*_*C*_ under carbon limitation has to be compensated by a reduction of the share of the rest of the proteome (Fig. 1a). In *E. coli*, the mass fraction of many biosynthetic proteins, from anabolism to protein synthesis, indeed decreases with the growth rate in an approximately linear fashion.^3,5,6^ For simplicity, let us assume that the proteome fraction devoted activities other than carbon-import, which we denote as *ϕ*_NC_ ≡ 1 − *ϕ*_C_, adjusts with the growth rate so that *ϕ*_NC_ = *ϕ*_NC,0_ + *ε*_NC_*μ*, with an offset term *ϕ*_NC,0_ and a slope *ε*_NC_ (see Supplementary Text, Note 1 for a more general formulation). When plugged in Eq. (1), this results in

2 $$\mu = \frac{{1 - \phi _{{\mathrm{NC}},0}}}{{\varepsilon _{{\mathrm{NC}}} + q \cdot w_C}}.$$

The term *ϕ*_NC,0_ includes both constitutively expressed, “unnecessary” proteins (not required to grow in the given medium but important in fluctuating conditions^32^) and the basal abundance of metabolic enzymes. The coefficient *ε*_NC_, governing the growth-dependent part of the proteome, depends instead on the expression level of metabolic enzymes as well as on the efficiency of the metabolic pathways, including energy generation and anabolic processes.^5,6,30^ Because changes in proteome allocation impact the efficiency of carbon utilization, *q* and *ε*_NC_ cannot be varied independently. This will lead to tradeoffs in Eq. (2). Under sever carbon shortage (high stress, large *w*_*C*_), *μ* is dominated by the carbon efficiency term, i.e., *μ* ∝ (*q* ⋅ *w*_*C*_)^−1^ as in (1), and maximum growth coincides with minimum *q*. In carbon-rich conditions (low stress, small *w*_*C*_), instead, *μ* ∝ 1/*ε*_NC_ and maximum growth is achieved by minimizing the proteome cost of the other metabolic activities.

Eq. (2) is purely phenomenological. We will now re-derive it within a genome-scale model of *E. coli* metabolism based on empirical growth laws.^4^ This will allow us to quantitatively describe the changes in cellular metabolism and proteome organization that occur under carbon limitation.

### Empirical growth laws quantitatively link growth rate, specific carbon intake, and enzyme costs

Multiple studies have shown that, in carbon-limited growth, *E. coli*’s proteome is partitioned into sectors whose relative sizes adjust with the growth conditions.^3,5,6^ For our purposes, the simplest partition involves four sectors, three responding to the growth condition [ribosome-affiliated proteins (*R*), metabolic enzymes (*E*) and proteins involved in the carbon-uptake system (*C*)], and the fourth with a fixed proteome share (‘housekeeping’ sector *Q*). Assuming proportionality to enzyme expression, metabolic fluxes effectively mediate proteome re-shaping.^3,5,30^ In this scenario the normalization of proteome mass fractions takes the form (Fig. 1b and Methods)

3 $$w_CJ_C + \mathop {\sum}\limits_{i \in E} w_i|v_i| + w_R\mu = \phi _{{\mathrm{max}}},$$

where *J*_*C*_ is the carbon intake flux, *v*_*i*_ is the flux of reaction *i*, and *ϕ*_max_ is a constant set by the baseline expression levels of all proteins. The terms on the left-hand side of (3) correspond to the condition-dependent *C*-, *E*- and *R*-sectors. In particular (see previous sections), *w*_*C*_*J*_*C*_ describes the proteome share devoted to the limited activity, with *w*_*C*_ quantifying the level of nutritional stress. The *R*- and *E*-sectors, instead, form the non-limited proteome. The coefficients *w*_*i*_ hence represent the ‘cost’ of each reaction *i* ∈ *E* in terms of the proteome share to be allocated to its enzyme per unit of net flux, while *w*_*R*_ quantifies the proteome share of ribosome-affiliated proteins per unit of growth rate.

Eq. (3) implies that increased nutrient stress (i.e. larger *w*_*C*_) causes a flux-mediated re-shaping of the cellular proteome that in turn affects the growth rate (Fig. 1c). *J*_*C*_ is related to *μ* by the specific intake rate *q* = *J*_*C*_/*μ*; likewise, we define the specific protein cost $\varepsilon = \mathop {\sum}\nolimits_{i \in E} w_i|v_i|/\mu$. Using these definitions one can isolate *μ* from (3), obtaining

4 $$\mu = \frac{{\phi _{{\mathrm{max}}}}}{{\varepsilon + w_Cq + w_R}}.$$

Eq. (4) is the quantitative counterpart of Eq. (2). As in the latter, the interplay between carbon and enzyme efficiency is controlled by the stress level *w*_*C*_, while *w*_*R*_ and *ϕ*_max_ are parameters that can be derived from experiments. Note that, by definition, *q* and *ε* do not depend separately on the flux vector **v** = (*J*_*C*_, {*v*_*i*_}) and on the growth rate *μ* but, rather, are functions of the ratio **v**/*μ* ≡ ***ξ***, a vector whose entries *ξ*_*i*_ give the contribution of reaction *i* to the cellular biomass in terms of mmol of metabolites transformed per gram of dry cell. Hence ***ξ*** characterizes the “metabolic state” of the cell, and the vector **v** = *μ****ξ*** can in turn be seen as a parametrization of metabolic fluxes in terms of the state ***ξ*** and of the growth rate *μ*. From the point of view of regulation, instead, the vector ***ξ*** represents a regulation program, prescribing the growth-dependence of enzyme mass fractions.

Note that this linear parametrization of the fluxes is correct at sufficiently fast growth, i.e., for *μ* ≳ 0.5/h (Methods). This range includes the acetate switch and is the condition on which we focus here. At slower rates, the approximation will deteriorate due to the presence of non-negligible maintenance fluxes (Methods).

### In silico states of maximum growth display a Pareto optimal tradeoff between specific intake and protein costs

Based on (4), maximizing *μ* is equivalent to minimizing the total cost

5 $${\cal{C}}({\boldsymbol{\xi }}) = \varepsilon ({\boldsymbol{\xi }}) + w_C\,q({\boldsymbol{\xi }}).$$

**Fig. 2:**
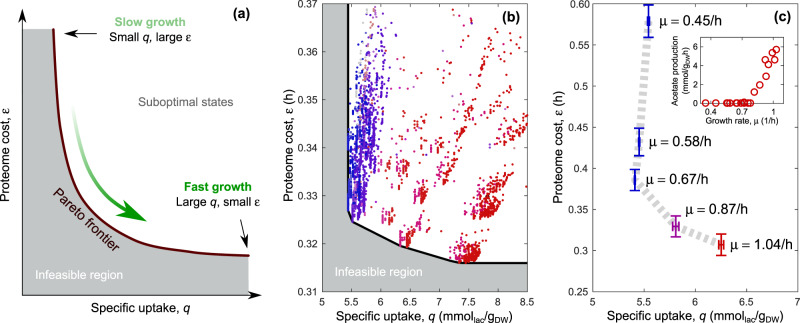
Tradeoff between minimum specific carbon intake and minimum enzyme cost in *E. coli*. **a** Multi-objective optimality and Pareto front. Two cost functions (specific carbon uptake *q* and specific proteome cost *ε*) are shown, together with the feasible (in white) and infeasible (in gray) regions, separated by the Pareto front. Optimal (i.e., growth maximizing) solutions lie on the latter. At slow growth, the optimal metabolic state corresponds to small *q* and large *ε*. As the growth rate increases (green arrow), optimal states move along the frontier, increasing *q* and reducing *ε*. **b** In silico prediction for optimal *E. coli* growth on lactose-limited minimal medium. The black line corresponds to the computed Pareto front, while individual points in the feasible region describe sub-optimal solutions. Blue (red) markers represent solutions dominated by respiration (fermentation), while purple markers denote mixtures. **c** *E. coli* states obtained by integrating mass spectroscopy data for lactose-limited growth from ref. ^6^ with in silico yields qualitatively reproduce (with quantitative accuracy for the yield) the predicted Pareto front. The values of *μ* reported next to the experimental points represent the experimental growth rates; colors represent the switch between respiration (in blue) and fermentation (in red), as determined experimentally (inset, data from Basan et al.^11^). Error bars represent the standard deviation

More precisely, for each value of *w*_*C*_, finding the flux vector **v** that maximizes *μ* subject to (3) is equivalent to finding the metabolic state ***ξ*** minimizing (5) (Supplementary Text Fig. S1). In other terms, the constraint (3) associates to each state ***ξ*** a growth rate *μ*(***ξ***) via (4). Such a *μ*(***ξ***) will be highest when ***ξ*** minimizes (5). Fluxes are then given by **v**(**ξ**) = **ξ***μ*(**ξ**) and satisfy the constraint (3) by construction. As *w*_*C*_ varies, cells must strike a compromise between the specific intake *q* and biosynthetic costs *ε* in order to maximize *μ*. The ensuing tradeoff between *q* and *ε* can be described by a Pareto front that separates an accessible region of the (*q*, *ε*) plane, such that each point lying therein represents a viable state ***ξ***, from an inaccessible one, with states of maximum growth lying on the front (Fig. 2a). *μ* increases as one moves along the Pareto front towards larger values of specific intake *q*, corresponding to lower growth yields.

Pareto-optimal and sub-optimal states for *E. coli* can be generated using a genome-scale model of metabolism (Fig. 2b, Methods and Supplementary Text, Note 2). Sub-optimal values of *q* and *ε* expectedly lie in the feasible region to the right of the Pareto front (black line). Markers in Fig. 2b, varying from blue (mostly respirative, lower *q*) to red (mostly fermentative, lower *ε*) show that *E. coli*’s metabolism is robustly characterized by respiration at slow growth and upregulated fermentation at fast growth, in agreement with experiments.

### Empirical data support Pareto-optimality

The above picture can be validated by estimating *ε* from mass spectrometry data^6^ and *q* from genome scale-modeling constrained by the empirical acetate overflow (Methods). The resulting curve in the (*q*, *ε*) plane (Fig. 2c) qualitatively agrees with the theory. At a quantitative level, the normalized protein cost *ε* predicted in silico for low-*q* states matches the observed enzyme cost at *μ* ≃ 0.7/h. For faster growth, where acetate excretion sets in, our model underestimates the decrease in *ε* by only about 10%, most likely due to the reduced efficiency of respiration enzymes.^11^ Likewise, at slow growth (below about 0.6/h) our prediction appears to underestimate *ε*, possibly due to the decrease in enzyme efficiencies that is known to set in at low *μ*^29,33–36^ and which is not accounted for in our constraint-based framework.

### The ATP yield is the key driver of the tradeoff

**Fig. 3:**
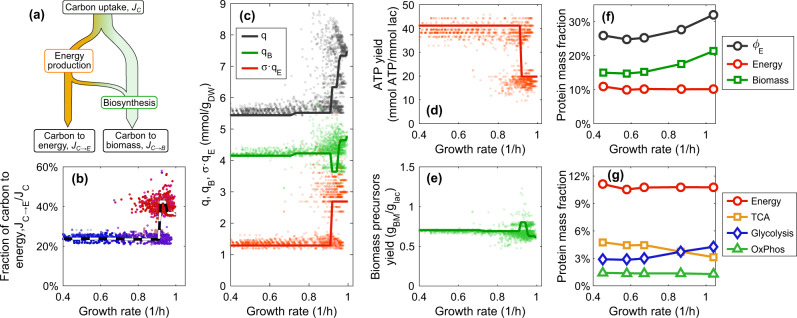
Carbon partitioning and the role of the ATP yield. **a** Scheme of the partitioning of the carbon intake flux into energy production and biomass precursors. Some of the energy produced by the cell is used for the synthesis of biomass precursors, and is hence accounted for in the overall precursors biosynthesis flux (carbon flux *J*_*C*→*B*_); the rest of the carbon (*J*_*C*→*E*_) is spent to generate the energy necessary for biosynthetic activities downstream of metabolism, e.g., translation. **b** The relative carbon-to-energy flux estimated by sampling a genome-scale metabolic model roughly varies between 25 and 40% of the overall carbon influx. Similarly to Fig. 2b, the color indicates the type of energetic metabolism (blue, respiration; red, fermentation), while the dashed line indicates the optimal solution corresponding to the Pareto front. **c** Behavior of the specific uptake *q* and of its biomass- (*q*_*B*_) and energy-related components (*σq*_*E*_), see Eq. (7). The change in *q* is mostly accounted for by the change in *σq*_*E*_. Solid lines represent the Pareto-optimal solution. **d** ATP yield, 1/*q*_*E*_, and **e** biomass yield as a function of *μ*. The sharp decrease in the former corresponds to the respiration-to-fermentation crossover leading to carbon overflow. Solid lines represent the Pareto-optimal solution. The biomass yield is given by the average molar mass of the biomass components, over *q*_*B*_. **f** Mass fractions of proteins associated to energy and biomass synthesis within the enzymatic sector (fraction *ϕ*_*E*_). **g** The respiration-to-fermentation crossover occurs within the energy-related mass fraction, which remains roughly constant as *μ* is changed. Proteomics data from ref. ^6^

The structure of metabolic states shown in Fig. 2b suggests a tight link between growth and energy metabolism, as fast (slow) growth favors the use of low (high) ATP yield pathways.^11^ Whether the efficiency of energy biosynthesis is the driver of carbon overflow is however not obvious from Eq. (5). In schematic terms (Fig. 3a), one can think that cells channel the in-taken carbon through pathways contributing respectively to the overall production of energy and to the synthesis of biomass precursors. However, about 1/3 of the energy production sustains anabolic processes underlying the synthesis of biomass building blocks (Supplementary Text Fig. S2), and therefore the corresponding carbon flux can also be associated to the biosynthesis of biomass precursors. Hence, we focus on the fluxes denoted respectively as *J*_*C*→*B*_ and *J*_*C*→*E*_ in Fig. 3a, corresponding to the carbon flux associated to biomass biosynthesis and the carbon flux associated to the production of energy feeding the cellular processes downstream of metabolism, e.g., protein synthesis, respectively. The two fluxes satisfy

6 $$J_C = J_{C \to B} + J_{C \to E},$$

with *J*_*C*_ being the total the carbon import rate. Based on (6), we can write the specific intake *q* = *J*_*C*_/*μ* as

7 $$q = q_B + \sigma q_E.$$

The quantity *q*_*B*_ = *J*_*C*→*B*_/*μ* represents the amount of carbon used to synthesize biomass precursors (in units of mmol of carbon per gram of dry weight). The term *q*_*E*_ = *J*_*C*→*E*_/(*σμ*) instead stands for the carbon intake associated to ATP production (i.e., the inverse number of ATP molecules regenerated per lactose molecule), where we accounted for the fact that at fast growth the overall energy requirement *J*_*E*_ of processes downstream of metabolism (e.g. translation) is proportional to the growth rate *μ* via a fixed growth-associated maintenance rate *σ*, i.e. *J*_*E*_ = *σμ* (Methods). To assess the partitioning of carbon into energy production and biomass precursors one has to estimate *q*_*E*_ and *q*_*B*_. This can be done using a genome-scale model of *E. coli*’s metabolic network upon generalizing the parametrization **v** = *μ****ξ*** to the linear combination **v** = *J*_*E*_***η*** + *μ****β***, where the vectors ***η*** and ***β*** can be univocally determined by perturbing the energetic demand of the cell (Supplementary Text, Note 3). In this setting, the metabolic state of the cell is described by the pair of vectors (***η***, ***β***) rather than by the single vector ***ξ***, but it is still possible to compute optimal and sub-optimal states together with the corresponding values of *q*_*B*_ and *q*_*E*_ (Supplementary Text, Note 3C).

Our results indicate that the carbon flux *J*_*C*_ is diverted preferentially into the synthesis of biomass precursors, with *J*_*C*→*B*_ amounting to 60–75% of *J*_*C*_ (Fig. 3b). Still, most of the variation in the specific intake *q* with *μ* is accounted for by a significant increase in *q*_*E*_ (Fig. 3c), corresponding to a dramatic decrease in ATP yield (1/*q*_*E*_, Fig. 3d) from 40 ATP per lactose for respiration-based states to about 20 for fermentation-based ones, in agreement with estimates from ref. ^11^ On the other hand, the biomass yield (1/*q*_*B*_, Fig. 3e) does not display any significant change. In other terms, the energy yield appears to be the main driver of the acetate switch despite the significantly uneven partitioning of carbon between ATP and biomass precursor synthesis pathways.

By exploiting the same decomposition of **v** we are also able to infer whether individual reactions are mostly associated to the synthesis of energy or biomass precursors. This information is encoded in the entries of ***η*** and ***β***, which relate fluxes respectively to the energy and the biomass class. Non-zero entries of ***η*** occur mostly for reactions belonging to glycolysis, TCA cycle, and oxidative phosphorylation pathways. The corresponding proteome mass fractions derived from proteomics sum up to about 11% of the total in all conditions (Fig. 3f), while the crossover between TCA and glycolytic proteins starts at growth rates faster than 0.7/h (Figs 3g and 2c, inset) in agreement with previous determinations.^11^ In turn, the mass fraction of proteins associated to the production of biomass precursors increases almost proportionally to the growth rate for *μ* ≳ 0.7/h (Fig. 3f), implying that the decrease in *ε* = *ϕ*_*E*_/*μ* observed at fast growth (Fig. 2c) is mostly due to the fact that the proteome share allocated to energy metabolism remains roughly constant as *μ* changes.

### A two-state model of *E. coli*’s acetate switch

**Fig. 4:**
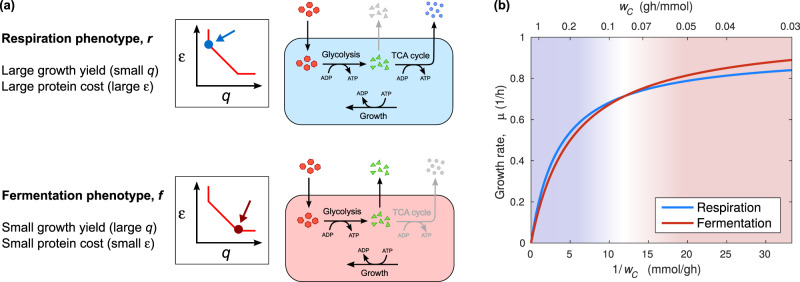
Phenomenological two-state view of *E. coli* carbon-limited growth. **a** Respiration and fermentation phenotypes as characterized by the multi-objective optimal states on the Pareto front of *E. coli*’s metabolism. The respiration phenotype has a larger yield (smaller *q*) and larger specific protein costs, while the fermentation phenotype carries lower yields (higher *q*) and smaller cost. **b** Growth rate (*μ*) versus carbon-intake cost *w*_*C*_ as obtained from the phenomenological two-state model discussed in the text (see Supplementary Text, Note 4). For each *w*_*C*_, the optimal phenotype is the one for which *μ* is largest. The switch from the fermentation to the respiration phenotype occurs when *w*_*C*_ matches the extra protein cost required by respiration

These results strongly implicate energy metabolism in the increase in the specific carbon uptake *q* and the corresponding drop in the protein cost *ε* observed at fast growth. The presence of two well-defined types of states (associated respectively to respiration and fermentation), the shift in the efficiency of the optimal energetic metabolism, and the lack of significant changes in the efficiency of anabolic processes point to a minimal two-state model of *E. coli*’s acetate switch. We classify states on the Pareto front in two broad sets (Fig. 4a). The first corresponds to a ‘fermentation’ phenotype with large specific carbon intake (*q*_fer_ ≳ 6 g_lac_/g_DW_, Fig. 2c) but low specific protein cost (*ε*_fer_ ≃ 0.3 h), showing carbon overflow and robust fermentative flux. The second one has lower carbon intake (*q*_res_ ≳ 5.5 g_lac_/g_DW_) but higher protein costs (*ε*_res_ ≃ 0.35 h) and relies on respiration for ATP production. We assume that generic flux patterns are described by linear combinations of these phenotypes with parameter *α* (0 ≤ *α* ≤ 1), thereby giving a combined specific carbon intake *q*(*α*) = *αq*_res_ + (1 − *α*)*q*_fer_ and protein cost *ε*(*α*) = *αε*_res_ + (1 − *α*)*ε*_fer_. The growth rate *μ* is then

8 $$\mu (\alpha ) = \frac{{\phi _{max}}}{{w_R + \varepsilon (\alpha ) + w_C\,q(\alpha )}}$$

(see Eq. (4)). This model predicts (Supplementary Text, Note 4) that, at optimal growth, a transition between the fermentation phenotype (fast growth) and the respiration phenotype (slow growth) occurs when the extra cost of importing carbon in the former matches the extra protein cost of respiration in the latter (Fig. 4b). The transition occurs when *w*_*C*_ takes the value

9 $$w_C^{{\mathrm{ac}}} \equiv \frac{{\varepsilon _{{\mathrm{res}}} - \varepsilon _{{\mathrm{fer}}}}}{{q_{{\mathrm{fer}}} - q_{{\mathrm{res}}}}} \simeq 0.1\frac{{{\mathrm{g}}_{{\mathrm{DW}}} \cdot {\mathrm{h}}}}{{{\mathrm{mmol}}\,{\mathrm{lac}}}},$$

corresponding to *μ*_ac_ ≃ 0.7/h, close to the experimentally determined onset of the acetate switch (Fig. 2c). While a discontinuous respiration-fermentation transition is unrealistic, this model emphasizes the role of the ATP yield and the interplay between specific carbon intake and protein costs in determining carbon overflow in *E. coli*. The value $w_C^{{\mathrm{ac}}}$ above which the fermentation phenotype becomes dominant and acetate excretion sets in can be interpreted as the external carbon level for which the two strategies provide the same cost-benefit balance, therefore giving the same growth rate. A comparison between this toy model and other phenomenological approaches is given in Supplementary Text, Note 4.

## Discussion

We have provided a description of *E. coli*’s carbon-limited growth in terms of a multi-objective optimization problem in which the cell optimizes for both the efficiency of carbon utilization and the protein cost of metabolism. As the external carbon level is varied, the optimal solution interpolates between the two functions, focusing the optimization on protein costs (carbon efficiency) in carbon-rich (carbon-poor) media. The emergent scenario depicts, with quantitative accuracy, the economics underlying the yield-cost tradeoff in *E. coli*. States associated to respiration or fermentation lie in different regions of the plane spanned by the specific carbon uptake *q* and the specific protein cost *ε*. Because of the proteome allocation constraint Eq. (3), a predominantly respirative, yield-maximizing strategy is optimal at slow growth, while a cost-minimizing strategy relying on fermentation sets in at fast growth. The Pareto front bends between *μ* ≃ 0.6/*h* and *μ* ≃ 0.9/*h*, signaling the gradual shift from one strategy to the other. Remarkably, this range contains the empirical acetate onset point, which lies at *μ* ≃ 0.7/h.^11^ To clarify which variable drives the change in growth yield observed along the crossover, we developed a genome-scale in silico framework to decompose the carbon influx into terms contributing respectively to biomass and energy synthesis. We found that the partitioning is significantly uneven, with most carbon going into biomass precursors. The energy-biosynthetic component, however, accounts for most of the change seen in the growth yield. The efficiency of ATP production hence appears as the key driver of the yield-cost tradeoff, and a minimal two-state model based on this result can indeed recapitulate the overflow scenario.

The analysis of optimal growth in terms of a yield-cost tradeoff and the decomposition of the carbon uptake into energy and biomass contributions are the main results of this work. When integrated with proteomic data, such a decomposition allows to comprehend the behavior of metabolic fluxes and proteome mass fractions in a unified framework. It is noteworthy that a detailed study of carbon partitioning had so far been carried out only within streamlined models.^37–40^

The two-state scheme presented here differs from other coarse-grained models of carbon overflow in many respects. For instance, the yields and cost parameters for respiration and fermentation used in ref. ^11^ refer to the ATP yield (as opposed to the growth yield) and to specific “respiration” and “fermentation” proteomes. Here, both pathways belong to the same *E*-sector and the focus is on the global re-allocation of the proteome rather than the up- or down-regulation of specific pathways. Secondly, the cost of carbon uptake (i.e., *w*_*C*_) is implicitly assumed to be nil in ref.,^11^ implying that metabolism is completely determined by the normalization of proteome fractions and by the carbon and energy flux balance. Under Pareto optimality, instead, it derives from the tradeoff between the *C*- and *E*-sectors. This makes it in principle possible to describe strains with different “acetate overflow lines” (e.g., mutants^41,42^ or “acetate feeding” strains obtained in evolution experiments^43,44^), which correspond to feasible (albeit sub-optimal) cellular states that would be harder to describe by the model of ref., ^11^ which characterizes an “optimal” strain. Experiments probing the fitness advantages of different metabolic strategies in different environmental and ecological contexts might shed light on the evolution of the acetate switch.

By extending the model of Basan et al.,^11^ Vazquez and Oltvai^45^ have linked overflow metabolism to a macromolecular crowding constraint along the lines of refs. ^46,47^ (A similar constraint plays an important role in the self-replicating cell model discussed in ref. ^22^) For *E. coli*, this scenario appears to be at odds with the empirical fact that the cell volume adjusts in response to changes in the macromolecular composition of the cell so as to maintain the density roughly constant across distinct conditions.^48,49^ While this reinforces the idea that cytoplasmic crowding is finely regulated,^50^ a constant intracellular density is unlikely to be the main driver of the acetate switch. In addition, however, ref. ^45^ points out that, at slow growth, an increase of the proteome share of proteins other than those associated to respiration and fermentation has to take place. Our results are in line with this observation. In fact, proteins included in the *C*-sector are upregulated at low *μ*, in agreement with quantitative measurements.^6^

It is known that many different organisms share *E. coli*’s behavior in terms of growth laws^4^ and carbon overflow. The detailed picture may, however, differ significantly from *E. coli*’s. For instance, recent work on *L. lactis*, an industrial bacterium that displays a transition between different types of fermentations rather than between fermentation and respiration, indicates that protein costs are not a determinant factor in its growth strategies.^51^ On the other hand, many features observed in *E. coli* are shared by *S. cerevisiæ*,^52^ despite the fact that carbon overflow in yeast appears to respond to the carbon intake flux rather than to the macroscopic growth rate.^53^ Likewise, relationships like Eqs. (1) and (2) hold under more general types of limitations than carbon shortage, provided *J*_*C*_ is replaced by the flux of the limited activity and *w*_*C*_ by a proxy for the level of stress to which the cell is subject. The tradeoff theory presented here is therefore likely to be more broadly applicable than the case study on which we have focused.

## Methods

### Derivation of the proteome constraint, Eq. (3)

The proteome normalization condition using the *C*-, *R*-, *E*- and *Q*-sectors reads

10 $$\phi _C + \phi _R + \phi _E = \underbrace {1 - \phi _Q}_{{\mathrm{fixed}}}.$$

Based on the bacterial ‘growth laws’ characterized in refs., ^3,6^ each of the three condition-dependent terms in Eq. (10) is of the form *ϕ*_*X*_ = *ϕ*_*X*,0_ + Δ*ϕ*_*X*_, where *ϕ*_*X*,0_ is a constant offset value, the minimal value of Δ*ϕ*_*X*_ is zero, and *X* ∈ {*C*, *R*, *E*}. In turn, if metabolism mediates the adjustment of each of the three Δ*ϕ*_*X*_ terms, these can be expressed in terms of their driving fluxes.^3,5,6,30^ In particular, using a linear enzyme-flux relation *ϕ*_*i*_ = *ϕ*_*i*,0_ + *w*_*i*_|*v*_*i*_| one gets to the following expressions for the condition-dependent parts of the proteome:

11 $$\begin{array}{*{20}{l}} {{\mathrm{\Delta }}\phi _C(J_C)} \hfill & = \hfill & {w_C\,J_C,} \hfill \\ {{\mathrm{\Delta }}\phi _R(\mu )} \hfill & = \hfill & {w_R\,\mu ,} \hfill \\ {{\mathrm{\Delta }}\phi _E({\mathrm{v}})} \hfill & = \hfill & {\mathop {\sum}\limits_{i \in E} w_i|v_i|.} \hfill \end{array}$$

Substituting these into Eq. (10) immediately leads to Eq. (3) upon including all condition-independent terms in the constant *ϕ*_max_ = 1 − *ϕ*_*C*,0_ − *ϕ*_*R*,0_ − *ϕ*_*E*,0_, where *ϕ*_*E*,0_ corresponds to the sum of the offsets of the individual metabolic enzymes: $\phi _{E,0} = \mathop {\sum}\nolimits_{i \in E} \phi _{i,0}$.

### Metabolic network reconstruction

All computations were carried out on the iJR904 GSM/GPR genome-scale metabolic model^54^ with a lactose-limited minimal medium.

### Constraint-based model to compute optimal metabolic fluxes

In order to compute reaction fluxes corresponding to maximum growth within a genome-scale model, we have resorted to Constrained Allocation Flux Balance Analysis (CAFBA). Flux Balance Analysis (FBA^55^) approaches to metabolic network modeling search for optimal flux vectors **v** = {*v*_*i*_} within the space ${\cal{F}}$ defined by the mass balance conditions **Sv** = **0**, **S** denoting the stoichiometric matrix, and by lower and upper bounds for the fluxes:

12 $${\cal{F}} = \left\{ {{\mathrm{v}}:{\mathrm{Sv}} = 0\,{\mathrm{and}}\,{\mathrm{v}}_{{\mathrm{min}}} \le {\mathrm{v}} \le {\mathrm{v}}_{{\mathrm{max}}}} \right\}.$$

The most common bounds encode for thermodynamic constraints imposing that *v*_*i*_ ≥ 0 for irreversible reactions. Other fluxes might instead be set to prescribed values, as the ATP hydrolysis flux related to maintenance (ATPm), which is fixed at 7.6 mmol/g_DW_h in the iJR904 model. FBA usually looks for the flux vector ${\mathrm{v}} \in {\cal{F}}$ that maximizes the growth rate *μ* subject to a cap on the nutrient intake, and is solved by Linear Programming (LP).^55^

CAFBA^30^ is a modification of FBA where ${\cal{F}}$ is further constrained by Eq. (3). CAFBA is explicitly stated as

13 $$\mathop{\mathrm{max}}\limits_{\mathrm{v} \in {\cal{F}}}\,\mu \quad {\mathrm{subj}}.\,{\mathrm{to}}\quad w_CJ_C + w_R\mu + \mathop {\sum}\limits_{i \in E} w_i|v_i| = \phi _{{\mathrm{max}}}.$$

As shown in ref., ^30^ CAFBA can be solved by LP as long as the protein constraint is growth-limiting. Importantly, no bounds on the influx of nutrients present in the growth medium (e.g., the carbon source) are required. In CAFBA, different degrees of limitation of the carbon source are modeled by increasing the value of *w*_*C*_, which is thus the main control parameter. We set *ϕ*_max_ and *w*_*R*_ to their *E. coli*-specific empirical values of 0.48 and 0.169/h, respectively.^3^ In addition, the costs *w*_*i*_ of reactions in the *E*-sector were assumed to take on the same value for each *i*, namely *w*_*E*_ = 8.3 × 10^−4^ g_DW_h/mmol.^30^ This value imposes a maximum achievable growth rate, obtained in the limit *w*_*C*_ → 0, of 1/h, comparable to that seen in.^6^ (See ref. ^30^ for a discussion of alternative choices.) For sakes of simplicity, we have also assumed that the stoichiometry of the biomass synthesis reaction whose rate quantifies *μ* is growth-rate independent.^55^ The case of growth-rate dependent biomass composition does not yield significant differences with the scenario we present.^30^

### Growth-associated and maintenance energy fluxes

In genome-scale models of metabolism, the energy requirements of the cell are described by an ATP hydrolysis flux *J*_*E*_ = *σ*_0_ + *σμ* formed by a growth-independent “maintenance” flux *σ*_0_ (the ATPm) and a growth-associated term *σμ*. These terms account for process not included in the metabolic network, like protein synthesis. In *E. coli*, the ratio *σ*_0_/*σ* is estimated to lie between 0.05 and 0.15/h.^30,56,57^ As a consequence, *σ*_0_ can be neglected at growth rates above roughly 0.5/h.

### Computation of the Pareto front

The Pareto front shown in Fig. 2b was computed by solving CAFBA for different values of *w*_*C*_ after silencing ATPm. To compensate for the lack of maintenance-associated energy costs, we increased the growth-associated ATP hydrolysis rate by an amount equal to the ATPm flux (i.e., 7.6 mmol_ATP_/g_DW_), so that the total ATP hydrolysis flux at the maximum achievable growth rate of 1/h is the same as in the default model. The difference in the overall ATP hydrolysis flux (including maintenance and growth-rate dependent components) between this implementation of CAFBA and the standard one^30^ is within 15% for growth rates above 0.5/h. For each CAFBA-derived optimal state **ξ** = **v**/*μ*, the specific intake *q* = *J*_*C*_/*μ* and the specific protein cost $\varepsilon = w_E\mathop {\sum}\nolimits_i |v_i|/\mu$ were computed, returning a set of points (one per state) in the (*q*, *ε*) plane. The Pareto front is obtained by joining all optimal states via straight lines; as *w*_*C*_ is varied, the optimal solution jumps between the states defining the front, leading to the discontinuities seen in Fig. 2b (see Supplementary Note 2 for mathematical details and an illustration of the optimal states in CAFBA). Such abrupt shifts are at odds with the smooth behavior observed in experiments^11^ and are due to our choice to work with at fixed weights *w*_*i*_.^30^ The version of CAFBA with randomized weights described in ref. ^30^ makes use of an averaging procedure that yields the same qualitative picture but quantitative agreement with experiments. Here we have chosen to stick to the non-random case for sakes of simplicity.

### Generation of sub-optimal CAFBA solutions

In order to generate the sub-optimal CAFBA solutions shown in Fig. 2b, we computed the values of *q* and *ε* for flux vectors **v** = (*J*_*C*_, {*v*_*i*_}) different from the optimal ones. To ensure that such states lie close to the Pareto front, we used flux vectors that are optimal for a version of CAFBA in which homogeneous costs (*w*_*i*_ = *w*_*E*_ for each *i*) are replaced by independent identically-distributed random costs with mean *w*_*E*_ and dispersion *δ*, as discussed in ref. ^30^ After generating a large number of such vectors for different values of *w*_*C*_, we computed the corresponding metabolic states ***ξ*** by normalizing each of them by its growth rate. For each such ***ξ***, we then computed the specific uptake *q* = *ξ*_*C*_ and protein cost $\varepsilon = w_E\mathop {\sum}\nolimits_i |\xi _i|$, using homogeneous weights for the latter. This method yields viable but sub-optimal solutions to CAFBA with homogeneous weights. The typical distance of sub-optimal solutions from the Pareto front is controlled by the dispersion *δ* of the individual costs *w*_*i*_.^30^ Sampled solutions approach the Pareto front as *δ* → 0. Upon increasing *δ*, protein costs get more variable and metabolic states far from the Pareto front become increasingly likely.

### Computation of ATP and biomass yields

Optimal and sub-optimal CAFBA solutions shown in Fig. 3 were generated as follows. For each solution **v** to CAFBA with randomized protein costs (obtained as described above), we computed a “perturbed” solution **v**′ using a small but non-zero ATPm (*σ*_0_ = 10^−2^ mmol ATP/g_DW_h). The vectors ***β*** and ***η***, in terms of which flux vectors can be decomposed, can be obtained uniquely as linear combinations of **v** and **v**′. The growth rate corresponding to a sub-optimal solution to the CAFBA problem with homogeneous proteins costs are instead computed from a generalization of Eq. (4) that includes an explicit dependence on *σ*_0_. Details are found in Supplementary Text, Note 3.

### Computation of protein costs from mass spectrometry data and comparison with the predicted Pareto front

Mass spectrometry data from^6^ quantify protein levels for *E. coli* NQ381 (a strain with titratable LacY enzyme derived from the prototrophic K-12 strain NCM3722) grown in minimal lactose media. Five different growth rates were obtained by inducing different levels of LacY. Relative protein abundances were converted into absolute mass fractions following the method used in.^11^ Briefly, we rescaled relative mass fractions with absolute abundances from ribosome profiling data obtained for *E. coli* strain MG1655^58^; strain-specific differences were accounted for by using the proteomics dataset from ref. ^8^ as a bridge between NCM3722 and MG1655. The specific cost *ε* of the *E*-sector shown in Fig. 2c was obtained as follows. First, reactions were assigned to the *E*-sector according to the partition used in ref. ^30^ Next, for each reaction, we obtained a corresponding list of enzymes from the Gene-Protein-Reaction matrix included in the iJR904 reconstruction. We denote by *n*_*i*,tot_ the number of reactions in which enzyme *i* participates (irrespective of whether they are assigned to the *E*-sector or not), and by *n*_*i*,*E*_ the number of such processes included in the *E*-sector. Given the experimental protein mass fractions *ϕ*_*i*_, our estimate for the mass fraction of the *E*-sector *ϕ*_*E*_, and its proteome cost *ε* are given respectively by

14 $$\phi _E = \mathop {\sum}\limits_{i \in E} \frac{{n_{i,E}}}{{n_{i,{\mathrm{tot}}}}}\phi _i\quad {\mathrm{and}}\quad \varepsilon = \frac{{\phi _E}}{\mu }.$$

Unfortunately, growth yields for these dataset are not available. Instead, these were estimated with FBA by minimizing the carbon intake flux while constraining the growth rates and acetate fluxes to their experimental values. Notice that yields may vary considerably across experiments, and that their value depends on those of maintenance and growth-dependent ATP hydrolysis rates. For our purposes, though, the key feature is the decrease that occurs at fast growth rates (above approx. 0.7/h) due to acetate excretion, which is robust and independent on the carbon source used.^11,30^

### Computation of energy- and biomass-associated proteome fractions

To provide a condition-independent partitioning of the protein mass fraction of the experimental *E*-sector into a biomass- and an energy-associated component we adopted a criterion that relies on the flux decomposition **v** = ***β****μ* + *J*_*E*_***η*** and on the whole set of sampled metabolic states shown in Fig. 3. Because of the inherent conceptual difficulty in disentangling the contribution of energy to biomass synthesis, our choice is rather conservative with respect to the biomass-associated proteome fraction. Specifically, a reaction is considered “energy-associated” (index *ε*) if the following conditions are satisfied simultaneously in at least one of the metabolic states shown in Fig. 3: (i) its flux *v*_*i*_ is significantly non-zero (specifically, |*v*_*i*_| > 10^−6^ mmol/g_DW_h); (ii) its *η*_*i*_ component is sufficiently large compared to *β*_*i*_ (specifically, |*η*_*i*_| > 0.1 ⋅ |*β*_*i*_|). Otherwise, it is “biomass-associated” (index ${\cal{B}}$). The biomass- and energy-associated proteome fractions are then defined as

15 $$\phi _E^{\cal{B}} = \mathop {\sum}\limits_{i \in {\cal{B}}} \frac{{n_{i,E}}}{{n_{i,{\mathrm{t}}ot}}}\phi _i\quad {\mathrm{and}}\quad \phi _E^\varepsilon = \mathop {\sum}\limits_{i \in \varepsilon } \frac{{n_{i,E}}}{{n_{i,{\mathrm{tot}}}}}\phi _i,$$

respectively. These quantities are shown in Fig. 3f along with $\phi _E = \phi _E^{\cal{B}} + \phi _E^\varepsilon$. The majority of proteins in *ε* belongs to basic energy-producing pathways (Fig. 3g). The pathway membership of each reaction was based on the “subsystems” description in the iJR904 model (‘Citric Acid Cycle’ for TCA cycle, ‘Glycolysis/Gluconeogenesis’ and ‘Pyruvate Metabolism’ for Glycolysis, and ‘Oxidative Phosphorylation’ for OxPhos). Notice that the size and composition of the energy sector *ε* resulting from our conservative classification is in agreement with that found in ref.,^11^ where reactions were subdivided manually.

### Reporting summary

Further information on research design is available in the Nature Research Reporting Summary linked to this article.

## Supplementary information

Reporting summary

Supplementary Text

## Data Availability

All data generated or analysed during this study are included in this article (and its Supplementary information files).

## Supplementary information

**Supplementary Information** accompanies the paper on the *npj Systems Biology and Applications* website (10.1038/s41540-019-0093-4).
