# Supplementary material for: A yield-cost tradeoff governs Escherichia coli’s decision between fermentation and respiration in carbon-limited growth
Source: NPJ Syst Biol Appl. 2019 May 1;5:16. doi: 10.1038/s41540-019-0093-4 (PMC6494807; doi:10.1038/s41540-019-0093-4)
Supplement: Supplementary file 2 — Supplementary Text [file 41540_2019_93_MOESM2_ESM.pdf]

A yield-cost tradeoff governs Escherichia coli's decision between fermentation and respiration in carbon-limited growth – SUPPLEMENTARY TEXT

Matteo Mori,[1] Enzo Marinari,[2,3,*] and Andrea De Martino[4,5,*]

[1]*Department of Physics, University of California, San Diego, CA, USA*

[2]*Dipartimento di Fisica, Sapienza Università di Roma, Rome, Italy*

[3]*INFN, Sezione di Roma 1, Rome, Italy*

[4]*Soft and Living Matter Lab, Institute of Nanotechnology (CNR-NANOTEC), Consiglio Nazionale delle Ricerche, Rome, Italy*

[5]*Italian Institute for Genomic Medicine, Turin, Italy*

* Co-last authors

CONTENTS

NOTE 1. Trade-offs in the general model of optimal protein allocation 3

A. Duality of maximum growth and optimal proteome allocation 3

B. Transitions between optimal phenotypes 5

C. Consequences for Constrained Allocation FBA 5

NOTE 2. Computation of the Pareto front 7

NOTE 3. Metabolic modes and flux decomposition 9

A. Definition and basic decomposition 9

B. More than one constraint: the case of ATP maintenance 10

C. Computation of energy and biomass vectors 11

D. Decomposition of carbon intake into energy- and biomass-associated components 12

E. Growth rate associated to each metabolic state 12

NOTE 4. Phenomenological model: definition and solution in different cases 14

SUPPLEMENTARY FIGURES 17

SUPPLEMENTARY TABLES 19

SUPPLEMENTARY REFERENCES 20

NOTE 1. TRADE-OFFS IN THE GENERAL MODEL OF OPTIMAL PROTEIN ALLOCATION

A. Duality of maximum growth and optimal proteome allocation

Here we consider in more detail the problem of characterizing the optimal strategy for the cell to reallocate its proteome when a specific metabolic activity L is limited. In the Main Text, we focus on L being the carbon uptake (C), but the same model can be in principle applied to other stress sources, e.g. limiting other nutrient sources (nitrogen, phosphate etc.) or antibiotics. In the following, μ stands for the growth rate, v_L for the flux of the limited activity (to be identified with the carbon import flux J_C under carbon limitation, see Main Text), $\phi_{NL} = 1 - \phi_L$ for the mass fraction of the rest of the proteome and $q = v_L/\mu$ for the specific flux of the limited activity (per unit of growth rate). These quantities in turn depend on internal variables of the cell such as metabolic fluxes and metabolite or enzyme levels (see e.g. [1]). However, instead of considering the whole space of states spanned by such variables, we will limit ourselves to a set of cellular states ("phenotypes") which we label with an index α. Each phenotype is assumed to be described by a different set of values for the limiting flux v_L and for ϕ_{NL} for each growth rate μ. We then define, for each α, the quantity

$$\Phi^\alpha(w_L, \mu) \equiv w_L v_L^\alpha(\mu) + \phi_{NL}^\alpha(\mu) \ , \tag{1}$$

where w_L denotes the cost of the protein controlling v_L^α (to be identified with the level of nutritional stress w_C under carbon limitation, see Main Text). Both v_L^α and ϕ_{NL}^α are modulated by the dilution rate (i.e. $\Phi^\alpha \equiv \Phi^\alpha(w_L, \mu)$). In particular, we make the natural assumption that both increase with μ for each α, namely

$$\frac{\mathrm{d}v_L^\alpha}{\mathrm{d}\mu} > 0 \quad , \quad \frac{\mathrm{d}\phi_{NL}^\alpha}{\mathrm{d}\mu} > 0 \qquad \forall \alpha \ . \tag{2}$$

For any given w_L, the growth rate $\mu^\alpha(w_L)$ pertaining to phenotype α can in principle be obtained by inverting the condition $\Phi^\alpha(\mu^\alpha, w_L) = 1$. The problem of growth rate maximization can thus be re-cast as the constrained maximization of μ^α over the index α for given w_L and $v^\alpha(\mu)$, i.e.

$$\alpha^\star = \arg\max_\alpha \mu \quad \text{subject to} \quad \Phi^\alpha(\mu, w_L) = 1 \ , \tag{3}$$

or, more simply,

$$\alpha^\star(w_L) = \arg\max_\alpha \mu^\alpha(w_L) \ . \tag{4}$$

with $\mu^{\alpha}(w_L)$ being a solution of Eq. (1). We call this the *direct proteome-constrained problem*. We denote by $v_L^\star$ and $\phi_{NL}^\star$ the values of v_L and ϕ_{NL} corresponding to $\alpha^\star$. One can easily show that, for any growth rate μ for which all these quantities exist,

$$v_L^{(q)} \leq v_L^\star \leq v_L^{(\varepsilon)} \tag{5}$$

$$\phi_{NL}^{(\varepsilon)} \leq \phi_{NL}^\star \leq \phi_{NL}^{(q)} \tag{6}$$

where $(v_L^{(q)}, \phi_{NL}^{(q)})$ and $(v_L^{(\varepsilon)}, \phi_{NL}^{(\varepsilon)})$ are the values of v_L and ϕ_{NL} respectively obtained from the solutions of

$$q\text{-problem} \quad : \quad \alpha^{(q)} = \arg\min_{\alpha} v_L^{\alpha}(\mu) \quad \text{s.t.} \quad \mu = \mu^{\alpha} \ , \tag{7}$$

$$\varepsilon\text{-problem} \quad : \quad \alpha^{(\varepsilon)} = \arg\min_{\alpha} \phi_{NL}^{\alpha}(\mu) \quad \text{s.t.} \quad \mu = \mu^{\alpha} \ , \tag{8}$$

where the values of μ are assumed to match the optimal growth rate in the direct formulation of the problem. In order to see this, one first has to introduce the following *dual* problem of the direct proteome-constrained problem (3):

$$\alpha^\star = \arg\min_{\alpha} \Phi^{\alpha}(\mu) \quad \text{subject to} \quad \mu = \mu^{\alpha} \ . \tag{9}$$

The solution of this problem is identical to the one of the direct problem, Eq. (3), provided $\mathrm{d}\Phi^\star/\mathrm{d}\lambda > 0$ and the growth rate μ is set so as to match the optimal one obtained in the direct problem (see [1]). Then, the proof is straightforward: because of the definitions of the optimization problems (8) and (9), $\phi_{NL}^{(\varepsilon)} \leq \phi_{NL}^\star$, and $w_L v_L^\star + \phi_{NL}^\star \leq w_L v_L^{(\varepsilon)} \phi_{NL}^{(\varepsilon)}$, respectively. The first inequality directly gives us one of the two constraints; another constraint, namely $v_L^\star \leq v_L^{(\varepsilon)}$, is obtained by using both inequalities. The demonstration of the remaining bounds (the q-bounds) is analogous.

Both the q- and the ε-problem can be shown to be equivalent to two other problems different from the direct proteome-constrained problem introduced above. Indeed, the former describes the minimization of the specific flux $q = v_L/\mu$ (or to the maximization of the growth yield, proportional to $1/q = \mu/v_L$) at fixed growth rate, while the latter is equivalent to the maximization of the growth rate subject to a constraint on ϕ_{NL}.

In other terms, optimal growth and optimal proteome allocation are dual to each other and solutions to the direct protein-constrained problem are bound to lie within a range defined by the q- and ε-problems. The existence of these bounds allows to study how cells may optimally handle different degrees of limitation, that is, different values of w_L, by switching between alternate

solutions. In particular, for $w_L = 0$, the solution to the original problem coincides with the solution to the ε-problem. As w_L increases, instead, the solution may shift towards states with larger yields (smaller specific rates) at the cost of increasing ϕ_{NL}.

B. Transitions between optimal phenotypes

For any given "phenotype" α, one can obtain a growth rate μ^α as a function of w_L by solving the equation $\Phi^\alpha(w_L, \mu) = 1$. Optimal phenotypes are those maximizing μ for each w_L. Assuming for simplicity that optimal states are unique, transitions between "phenotypes" α and β may occur at values of w_L such that $\mu^\alpha(w_L) = \mu^\beta(w_L)$ and $\mathrm{d}\mu^\alpha/\mathrm{d}w_L \neq \mathrm{d}\mu^\beta/\mathrm{d}w_L$. Since $\Phi^\alpha = \Phi^\beta = 1$, we would have

$$w_L(v_L^\alpha - v_L^\beta) = \phi_{NL}^\beta - \phi_{NL}^\alpha \ , \tag{10}$$

i.e. any decrease in the flux v_L has to be matched by an increase in the non-limited proteome ϕ_{NL}, highlighting the tradeoff between optimal proteome allocation and efficient use of limited resources. By differentiating Φ^α with respect to w_L one gets

$$\frac{\mathrm{d}\mu^\alpha}{\mathrm{d}w_L} = -\frac{v_L^\alpha}{\mathrm{d}\Phi^\alpha/\mathrm{d}\mu} \ , \tag{11}$$

and therefore, assuming $\mathrm{d}\mu^\alpha/\mathrm{d}w_L > \mathrm{d}\mu^\beta/\mathrm{d}w_L$,

$$\frac{v_L^\alpha}{\mathrm{d}\Phi^\alpha/\mathrm{d}\mu} < \frac{v_L^\beta}{\mathrm{d}\Phi^\beta/\mathrm{d}\mu} \ . \tag{12}$$

This equation not only involves the absolute magnitude of the limited flux v_L, but also the variation of the proteome as the growth rate changes.

C. Consequences for Constrained Allocation FBA

The consequences of equations (10) and (12) depend on the model at hand. Let us consider, as in the main text, a flux-based constraint based model with proteome allocation constraint such as CAFBA, with no maintenance ATP hydrolysis rate. In this case, as explained in the Main Text, we can introduce the metabolic states $\boldsymbol{\xi}$ and express the fluxes as $\mathbf{v} = \boldsymbol{\xi} \cdot \mu$. Let us consider a set of different metabolic states, identified by an index α as $\boldsymbol{\xi}^\alpha$. For each state we can compute the specific uptakes q^α and protein costs ε^α, so that $v_L^\alpha = q^\alpha \mu$ and $\phi_{NL}^\alpha = \varepsilon^\alpha \mu$. The bounds Eq. (5)

and (6) take the form

$$q^{(q)} \leq q^{\star} \leq q^{(\varepsilon)} \tag{13}$$

$$\varepsilon^{(\varepsilon)} \leq \varepsilon^{\star} \leq \varepsilon^{(q)} \quad , \tag{14}$$

where (q) and (ε) label to specific states $\boldsymbol{\xi}^{(q)}$ and $\boldsymbol{\xi}^{(\varepsilon)}$, while the asterisk indicates the optimal state. On the other hand, Equations (10) and (12) respectively become

$$w_L(q^{\alpha} - q^{\beta}) = \varepsilon^{\beta} - \varepsilon^{\alpha} \tag{15}$$

$$\frac{q^{\alpha}\mu}{w_L q^{\alpha} + \varepsilon^{\alpha}} < \frac{q^{\beta}\mu}{w_L q^{\beta} + \varepsilon^{\beta}} \quad \Rightarrow \quad \frac{\varepsilon^{\alpha}}{q^{\alpha}} > \frac{\varepsilon^{\beta}}{q^{\beta}} \quad . \tag{16}$$

Together, these two constraints imply that, at *any* transition between optimal states, both q and ε vary, and they do so in opposite directions. When $w_L = 0$, $\varepsilon^{\star} = \varepsilon^{(\varepsilon)}$, while the specific flux $q^{\star}$ is maximum. As w_L increases, at each transition q decreases as ε increases. These properties lie at the basis of the Pareto front analysis.

NOTE 2. COMPUTATION OF THE PARETO FRONT

Let us consider a value $\overline{w}_C$ of w_C such that states α and β with $\mathcal{C}^\alpha = q^\alpha w_C + \varepsilon^\alpha$ and $\mathcal{C}^\beta = q^\beta w_C + \varepsilon^\beta$ are optimal for $w_C = \overline{w}_C$, with $\mathcal{C}^\alpha = \mathcal{C}^\beta$ when $w_C = \overline{w}_C = (\varepsilon^\alpha - \varepsilon^\beta)/(q^\beta - q^\alpha)$. We consider for definiteness $q^\alpha > q^\beta$. Starting from $w_C < \overline{w}_C$ (so with β as the optimal state) we are interested in constraining the parameters of a sub-optimal state γ with cost $\mathcal{C}^\gamma$, such that $\mathcal{C}^\gamma > \mathcal{C}^\alpha$ for $w_C > \overline{w}_C$ and $\mathcal{C}^\gamma > \mathcal{C}^\beta$ for $w_C < \overline{w}_C$. In what follows, we assume that $q^\beta < q^\gamma < q^\alpha$.

Suppose that $w_C < \overline{w}_C$. The constraint $\mathcal{C}^\gamma > \mathcal{C}^\beta$ can be rewritten as

$$\begin{aligned}\varepsilon^\gamma > & w_C(q^\beta - q^\gamma) + \varepsilon^\beta \\ & = w_C(q^\beta - q^\gamma) + \varepsilon^\alpha + \overline{w}_C(q^\alpha - q^\beta) \\ & > \overline{w}_C(q^\beta - q^\gamma) + \varepsilon^\alpha + \overline{w}_C(q^\alpha - q^\beta) \\ & = \varepsilon^\alpha - \overline{w}_C(q^\gamma - q^\alpha) \ ,\end{aligned} \tag{17}$$

where we used $w_C(q^\beta - q^\gamma) > \overline{w}_C(q^\beta - q^\gamma)$ (since $w_C < \overline{w}_C$ and $q^\beta < q^\gamma$). Similarly, when $w_C > \overline{w}_C$, one gets

$$\epsilon^\gamma > \varepsilon^\beta - \overline{w}_C(q^\gamma - q^\beta) \ . \tag{18}$$

Note that conditions (17) and (18) identify the same half-space in the $(q^\gamma, \varepsilon^\gamma)$ plane, defined by the line passing through the points $(q^\alpha, \varepsilon^\alpha)$ and $(q^\beta, \varepsilon^\beta)$. Therefore, given a set of optimal states $\alpha, \beta, \ldots$, the Pareto frontier is obtained by connecting neighboring points with straight lines, as illustrated with a concrete example in Fig. N1.

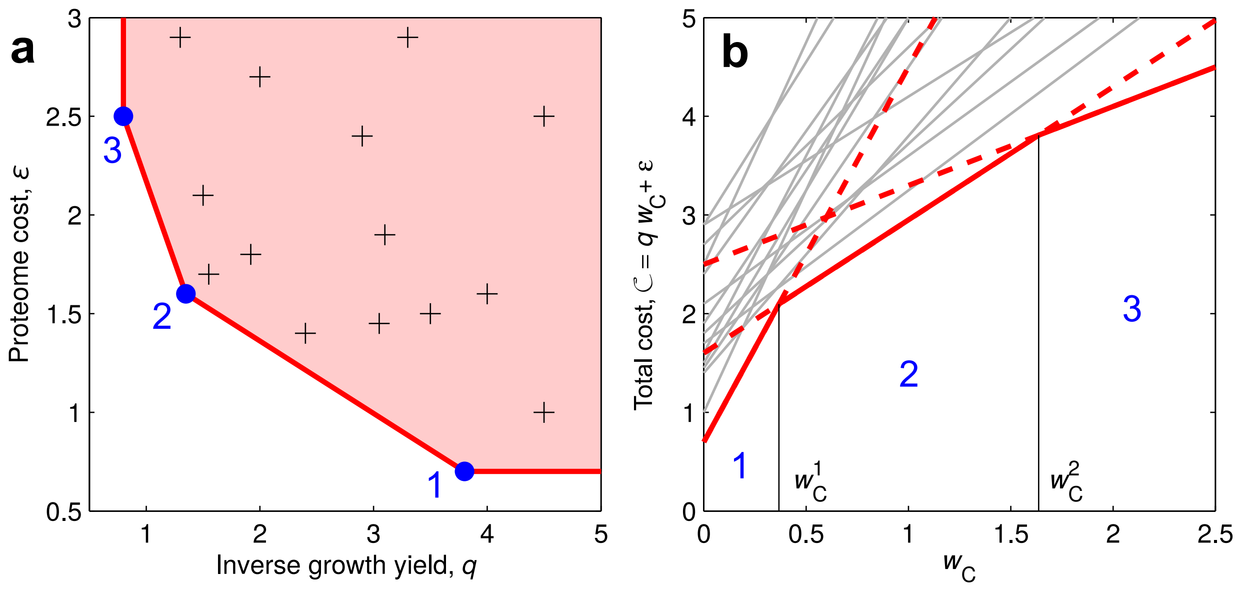

FIG. N1. Example of Pareto–optimality in CAFBA. **(a)**: Set of metabolic states in the (q, ε) plane. Each cross represents a suboptimal pathway, while the blue dots represent three Pareto-optimal metabolic states, numbered from one (minimum proteome cost ε^1) to three (minimum specific intake q^3). The Pareto frontier is shown in red and delimitates the feasible region (in light red). **(b)**: Total cost $\mathcal{C}^\alpha = q^\alpha w_C + \varepsilon^\alpha$ for each metabolic state α shown in panel (a). Each total cost $\mathcal{C}^\alpha$ is a linear function of w_C. The cost is minimized by the envelope (shown as a red line) of the lines corresponding to the three Pareto-optimal states (dashed lines). As w_C increases, the optimal metabolic mode switches from 1 to 2 at $w_C^1 = (\varepsilon^1 - \varepsilon^2)/(q^2 - q^1)$ and from 2 to 3 at $w_C^2 = (\varepsilon^2 - \varepsilon^3)/(q^3 - q^2)$. The grey lines denote the costs $\mathcal{C}^\alpha$ of suboptimal pathways (grey crosses in panel (a)).

NOTE 3. METABOLIC MODES AND FLUX DECOMPOSITION

A. Definition and basic decomposition

In this section we shall address in greater detail the definitions of metabolic states and the flux decomposition given in the Main Text, Eq. (6). In particular, we will study how the solutions of FBA-like problems depend on the parameters of the problem itself. To do so, it is useful to start from a "traditional" FBA setting in which the only constraints are (i) the stoichiometric constraints, $S\mathbf{v} = 0$; (ii) irreversibility constraints, $v_i \geq 0$ for some i; (iii) a constraint on the carbon intake flux, J_C (equivalently, it can also be an upper bound). For now, we will set the ATP maintenance flux σ_0 to zero. Given these constraints, we focus on flux configurations maximizing some linear functional of the fluxes; to be definite, we will simply pick the biomass synthesis flux μ. Also, for simplicity, in this note we will measure growth in terms of biomass accumulation instead of growth rate, i.e. μ has the same units of all other fluxes (mmol/g_{DW}h), so that the stoichiometric coefficients describing the biomass composition are dimensionless.

Purely by dimensional analysis, we see that the optimal solution $\mathbf{v}^\star$ to the FBA problem has to be proportional to the only unit-bearing quantity J_C, i.e.

$$\mathbf{v}^\star(J_C) = \boldsymbol{\xi} J_C \ , \tag{19}$$

where $\boldsymbol{\xi}$ is a dimensionless vector that implicitly depends on the network topology and on the active irreversibility bounds of the problem at hand, and it can be computed by dividing the optimal flux solution $(v)^\star$ by the carbon intake flux J_C. Eq. (19) simply states that the optimal fluxes are all proportional to each other upon varying J_C, as can be easily verified numerically. By specializing Eq. (19) to the biomass synthesis flux we obtain the relationship between carbon intake and growth rate, $J_C = \mu/\xi_\mu$; using this expression we can formulate an expression analogous to Eq. (19) expressing the fluxes as a function of the growth rate, $\mathbf{v}^\star(\mu) = \boldsymbol{\xi}\mu$ after redefining $\boldsymbol{\xi}/\xi_\mu \to \boldsymbol{\xi}$. Let us stress that these are *functional* relations: once both the vector $\boldsymbol{\xi}$ and a single flux, e.g. the growth rate μ, are known, the optimal solution is obtained *as a function of* μ as $\mathbf{v}^\star(\mu) = \boldsymbol{\xi} \cdot \mu$. The uniqueness of the $\boldsymbol{\xi}$ vector is linked to that of the FBA problem; if multiple solutions exist, then the vector $\boldsymbol{\xi}$ has the same degeneracy.

In the case of CAFBA there is not upper bound or constraint on the carbon intake flux. Instead, the protein constraint (Main text Eq. (3)) is responsible for bounding the magnitude of the optimal fluxes. By dividing both sides of Eq. (3) by w_C and splitting the fluxes into forward and backward

fluxes [2], we can rewrite the proteome constraint as $\mathbf{s} \cdot \mathbf{v} = z_C$, where the components of s are dimensionless ($s_i = w_i/w_C$) and $z_C = \phi_{\max}/w_C$ has units of flux. Again, because of the linearity of the problem, all optimal solutions can be written as a linear function of z_C as $\mathbf{v}^\star = \boldsymbol{\xi} z_C$. This time, however, $\boldsymbol{\xi}$ does in principle depend on the vector s and, thus, on w_C. Because of $\boldsymbol{\xi}$ being dimensionless, we must have $\mathrm{d}\boldsymbol{\xi}/\mathrm{d}w_C = 0$, which implies that $\boldsymbol{\xi}$ is, at most, a piecewise function of w_C (or z_C, or μ). Within each interval in which $\boldsymbol{\xi}$ is constant, the optimal solution will still be described by a linear function $\mathbf{v}^\star = \boldsymbol{\xi}\mu$. This is in agreement with the analysis presented in the Main Text, with the optimal solution ”jumping” between different metabolic states $\boldsymbol{\xi}$. Note that CAFBA solutions are mostly unique [2], and hence so is $\boldsymbol{\xi}$.

B. More than one constraint: the case of ATP maintenance

The main assumption behind Eq. (4) in the Main Text is that, for a given metabolic state $\boldsymbol{\xi}$, then $\mathbf{v} = \boldsymbol{\xi}\mu$ represents a valid set of fluxes, satisfying all stoichiometric and irreversibility constraints (not the proteome constraint, which is instead used to set the value of μ through Eq. (4)). If an ATP maintenance term is present, then this parametrization fails. Using again ”standard FBA” to simplify the discussion, but this time with a finite ATP maintenance flux, we see that there are two unit-bearing constants in the optimization problem, the carbon flux J_C and the ATP maintenance flux σ_0. Because of the linearity of the problem, the optimal solutions have to be first order functions of these two quantities, $\mathbf{v}^* = \boldsymbol{\xi}^{(1)} J_C + \boldsymbol{\xi}^{(2)}\sigma_0$. Note that a zero-order term is excluded by dimensional analysis. As before, the vectors $\boldsymbol{\xi}^{(1)}$ and $\boldsymbol{\xi}^{(2)}$ are uniquely determined if as the solution to the linear programming problem is also unique.

Even without proteome constraint, the two vectors $\boldsymbol{\xi}^{(1)}$ and $\boldsymbol{\xi}^{(2)}$ can be piecewise constant, although the flux themselves $v^\star$ are found to be continuous in FBA solutions. These discontinuities happen when some flux ”hits” an irreversibility constraint, and a new equality constraint is effectively introduced. This phenomenon has two main consequences. First, it is clear that these jumps can happen only when either $\boldsymbol{\xi}^{(1)}$ or $\boldsymbol{\xi}^{(2)}$ do not to satisfy the irreversibility constraints, even though they still satisfy the mass-balance constraints. This means that they cannot be considered, in isolation, a valid flux vector. Second, consider $\mathbf{v}(J_C, \sigma_0) = \boldsymbol{\xi}^{(1)} J_C + \boldsymbol{\xi}^{(2)}\sigma_0$ with fixed vectors $\boldsymbol{\xi}^{(1)}$ and $\boldsymbol{\xi}^{(2)}$. This function is not guaranteed to satisfy the irreversibility constraints for arbitrary values of J_C and σ_0. On the other hand, it is in possible to compute this function for any optimal solution $\mathbf{v}^\star$, and the resulting function could be used to provide vectors in at least a neighborhood

of the optimal solution, i.e. for J_C and σ_0 not far from the values corresponding to the ones used to compute $\mathbf{v}^\star$.

With the proteome constraint instead of the bound on carbon uptake, the same results apply, except one has z_C instead of J_C, and both ”continuous” (due to the irreversibility constraints) and ”discontinuous” (due to the proteome constraint) jumps are present in the two metabolic vectors. By trading J_C for the growth rate μ, and σ_0 for the total energy flux $J_E = \sigma_0 + \sigma\mu$, we can express the optimal solution as a linear combination of μ and J_E, i.e.

$$\mathbf{v}^\star(\mu, J_E) = \boldsymbol{\beta}\mu + \boldsymbol{\eta} J_E \ . \tag{20}$$

C. Computation of energy and biomass vectors

The vectors $\boldsymbol{\beta}$ and $\boldsymbol{\eta}$ used to compute the ATP and biomass yields in Fig. 3 were computed as follows. We first assume that the optimal solution $\mathbf{v}^\star$ of a CAFBA problem (with either uniform or randomized E-sector weights) can be *locally* parametrized as a function of growth rate μ and total energy flux J_E as described in Eq. (20). In particular, this expression will be valid for the optimal solution of a CAFBA problem, which we indicate with a star:

$$\mathbf{v}^\star = \boldsymbol{\beta}\mu^\star + \boldsymbol{\eta} J_E^\star \ . \tag{21}$$

This expression provides a constraint between the two vectors $\boldsymbol{\beta}$ and $\boldsymbol{\eta}$. The other constraint is obtained by computing a perturbed solution, obtained by solving CAFBA with all the same parameters as before except for the ATP maintenance flux, which is slightly increased (e.g. by 10^{-2} mmol ATP/g_{DW}h). As long as the perturbation is small enough, this solution is related to the (perturbed, indicated by the tilde) growth rate and energy flux through the same metabolic modes $\boldsymbol{\beta}$ and $\boldsymbol{\eta}$:

$$\tilde{\mathbf{v}}^\star = \boldsymbol{\beta}\tilde{\mu}^\star + \boldsymbol{\eta} \tilde{J_E}^\star \ . \tag{22}$$

Equations (21)-(22) can then be easily inverted to find the vectors $\boldsymbol{\beta}$ and $\boldsymbol{\eta}$ by solving a two-dimensional linear system for each reaction:

$$\begin{bmatrix} \beta_i \\ \eta_i \end{bmatrix} = \begin{bmatrix} \mu^\star & J_E^\star \\ \tilde{\mu}^\star & \tilde{J_E}^\star \end{bmatrix}^{-1} \begin{bmatrix} v_i^\star \\ \tilde{v}_i^\star \end{bmatrix} \ . \tag{23}$$

Of course, the flux components β_i and η_i cannot be uniquely determined if $v_i^\star = \tilde{v}_i^\star = 0$, and we thus set both of them to zero. Repeated optimization of CAFBA with randomized E-sector protein

costs provides a sampling of different metabolic states, i.e. pairs $(\boldsymbol{\beta}, \boldsymbol{\eta})$. We checked numerically that both $\boldsymbol{\beta}$ and $\boldsymbol{\eta}$ are independent on the perturbation applied. For instance, slightly perturbing w_C instead of σ_0 yields the same vectors.

D. Decomposition of carbon intake into energy- and biomass-associated components

The decomposition $J_C = J_{C\to E} + J_{C\to B}$ (Main Text Eq. (6)) is obtained by specializing Eq. (20) to the case of carbon intake, $v_i = J_C$. The right side of the equation becomes:

$$J_C = \beta_C \mu + \eta_C J_E \tag{24}$$

$$= \left(\beta_C + \eta_C \frac{J_E}{\mu}\right) \mu \tag{25}$$

$$= \left(\beta_C + \sigma \eta_C\right) \mu \ , \tag{26}$$

where we neglected for simplicity the growth-independent ATP maintenance σ_0 (the same approximation used throughout the Main Text). Therefore we obtain Eq. (7) from the Main Text:

$$q \equiv \frac{J_C}{\mu} = q_B + \sigma q_E \tag{27}$$

where we defined $q_B \equiv \beta_C$ and $q_E \equiv \eta_C$. We see that q_E is the specific carbon uptake per ATP produced, i.e. $1/q_E$ is the ATP yield per lactose molecule. Instead, $1/q_B$ is the biomass yield per lactose molecule, in units of $\mathrm{g_{DW}/mmol_{lac}}$; it is easily converted to $\mathrm{g_{\mathbf{DW}}/g_{lac}}$ units by dividing q_B by the specific mass of lactose.

E. Growth rate associated to each metabolic state

In this section we will show how to compute a flux vector $\mathbf{v}$ as a function of the growth rate and the ATP maintenance flux, starting from the metabolic modes $(\boldsymbol{\beta}, \boldsymbol{\eta})$ calculated above. This can be done in full generality by including a nonzero ATP maintenance flux σ_0.

The starting point is the parametrization of fluxes as a function of the growth rate μ and of the energy flux J_E given by Eq. (20). Since the energy flux and the growth rate are related by $J_E = \sigma_0 + \sigma\mu$, it is more useful in the following to express the optimal fluxes as a function of μ and σ_0:

$$\mathbf{v}^\star(\mu, \sigma_0) = \boldsymbol{\xi}\mu + \boldsymbol{\eta}\sigma_0 \ , \tag{28}$$

where we defined $\boldsymbol{\xi} = \boldsymbol{\beta} + \sigma\boldsymbol{\eta}$. Similarly to the case of vanishing σ_0, we define the specific intake fluxes $q = \xi_C$ and $q_0 = \eta_C$. For the E-sector protein costs, we have the additional problem of having to separate the absolute value of each flux, $|v_i|$, into the sum of two terms which might not have the same sign. While it impossible to obtain a simple expression for arbitrary vectors $\boldsymbol{\xi}$ and $\boldsymbol{\eta}$, it is feasible if we restrict ourselves to small variations in μ and σ_0 from the values obtained during the sampling procedure. We define s_i as the sign of the optimal flux $v_i^\star$ used above to compute the two vectors $\boldsymbol{\xi}$ and $\boldsymbol{\eta}$. Then, we write:

$$\begin{aligned} w_E \sum_i |v_i| &= w_E \sum_i s_i v_i \\ &= w_E \sum_i s_i \left(\xi_i \mu + \eta_i \sigma_0\right) \\ &= \sum_i \left[(w_E s_i \xi_i)\mu + (w_E s_i \eta_i)\sigma_0\right] \\ &\equiv \varepsilon\mu + \varepsilon_0\sigma_0 \ . \end{aligned}$$

This expression is valid in a neighborhood of the optimal solution $\mathbf{v}^\star$, as long as all fluxes keep the same sign upon varying μ and σ_0. Using the proteome constraint, one obtains the following generalization of Eq. (4) from the Main Text:

$$\mu = \frac{\phi_{\max} - (q_0 w_C + \varepsilon_0)\sigma_0}{q w_C + \varepsilon + w_R} \tag{29}$$

If we let $\sigma_0 \to 0$, the flux decomposition Eq. (28) reduces to $\mathbf{v} = \boldsymbol{\xi}\mu$, and Equation (29) reduces to Main Text Eq. (4); after obtaining μ, one can then compute the fluxes $\mathbf{v}$ from Eq. (28). Finally, one has to check whether some flux changed sign with respect to s_i, in which case one has to discard the solution. (We observe a very small rejection rate, about 1%.) Both vectors $\boldsymbol{\beta}$ and, in particular, $\boldsymbol{\eta}$ are well defined in the limit $\sigma_0 \to 0$, the reason being that the decomposition of the fluxes into energy- and biomass-associated components can be performed irrespective of the precise functional relationship between μ and $J_E(\mu)$.

The optimality of the pair $(\boldsymbol{\xi}, \boldsymbol{\eta})$, or equivalently $(\boldsymbol{\beta}, \boldsymbol{\eta})$, depends now on four variables, two specific fluxes (q and q_0, or q_B and q_E) and two protein costs (ε and ε_0, or ε_B and ε_E), and therefore the Pareto optimality of the solutions with finite σ_0 is harder to evaluate. As $\sigma_0 \to 0$, the Pareto optimality between q and ε is recovered, with q monotonously decreasing as w_C is increased. However, this does not constrain the specific fluxes for energy q_E and biomass q_B to be monotonously related to w_C; in fact the optimal q_B observed in Main Text Fig. 3 is a non-monotonic function of the growth rate.

NOTE 4. PHENOMENOLOGICAL MODEL: DEFINITION AND SOLUTION IN DIFFERENT CASES

We consider a simple reaction network (see Fig. N2a) involving 5 reactions among 3 chemical species, two of which (m_1 and m_2) can be exchanged with the exterior. Production or consumption of an "energy" metabolite e represents synthesis or hydrolysis of ATP. Reactions are summed up as

$$\begin{array}{rl} \text{“glycolysis” (rate } g) & m_1 \to a \cdot m_2 + b_1 \cdot e \\ \text{“respiration” (rate } r) & m_2 \to b_2 \cdot e \\ m_1 \text{ import (rate } u) & \emptyset \to m_1 \\ m_2 \text{ export (rate } v) & m_2 \to \emptyset \\ \text{Energy consumption, growth (rate } \mu) & e \to \emptyset \end{array} \tag{30}$$

Here growth is proportional to the consumption of the metabolite e and, for simplicity, we use the same units for both. Under steady-state mass-balance, one has $u = g$, $au = r + v$ and $\mu = b_1 g + b_2 r$. Flux states compatible with these constraints can be expressed as functions of μ and u alone. From $r \geq 0$ and $v \geq 0$ one gets instead the following bounds for the growth rate,

$$b_1 u \leq \mu \leq (b_1 + ab_2)u \ , \tag{31}$$

or, introducing the growth yield $Y \equiv \mu/u$ (reciprocal of the specific influx, $Y \equiv 1/q$):

$$b_1 \leq Y \leq b_1 + ab_2 \ . \tag{32}$$

Equation (32) implies that the different steady states are characterized by yields between the yield of fermentation $Y_{\text{fer}} = b_1$ and that of respiration $Y_{\text{res}} = b_1 + ab_2 > Y_{\text{fer}}$. As Eq. (31) does not by itself limit the growth rate, extra constraints have to be enforced to obtain well-defined solutions to the problem of maximizing μ. The nature of optimal states therefore depends on which additional constraints are imposed. We consider three distinct scenarios, whose solutions are summarized in Fig. N2b–d.

Standard FBA scenario: A minimal choice consists in imposing an upper bound to the carbon uptake flux, $u \leq u_{\max}$, where $u_{\max}$ may coincide with the maximum carbon uptake rates observed in experiments with continuous cultures. In this condition, maximization of μ

returns the state with the largest growth yield, i.e. $\mu^\star = Y_{\text{res}} u_{\max}$, $r^\star = a u_{\max}$ and $v^\star = 0$. This is a well known property of the solutions of standard FBA.

FBA with Molecular Crowding (FBAwMC) scenario [3, 4]: In this case a "crowding constraint" is imposed, consisting of an overall bound on intracellular fluxes of the form $c_1 g + c_2 r = 1$. Now, for the growth rate one finds $\mu = b_2/c_2 + (b_1 - b_2 c_1/c_2) g$. For $b_1/c_1 > b_2/c_2$, the optimal solution is obtained by minimizing g, i.e. by setting $g = g^\star \equiv (b_1 + a b_2)^{-1}\mu$, and presents respiratory metabolism. If instead $b_1/c_1 < b_2/c_2$, then g should be maximized ($g^\star = \mu/b_1$) and the optimal solution presents fermentative metabolism. The inclusion of explicit coefficients for the carbon uptake u and the fermentation flux v in the crowding constraint only leads to a re-definition of the coefficients c_1 and c_2.

Constrained Allocation FBA (CAFBA) scenario [2]: In this case, the additional constraint models proteome allocation and reads $w_C u + w_1 g + w_2 r + w_R \mu = \phi_{\max}$. As in the FBAwMC case, the growth rate can be expressed as a function of u alone, obtaining

$$\mu = \frac{1}{w_2/b_2 + w_R}\Big[\phi_{\max} + (Q - w_C)\cdot u\Big] \tag{33}$$

with $Q = b_1 \cdot [(w_2/b_2) - (w_1/b_1)]$. Q is proportional to the additional protein cost of respiration, w_2/b_2, with respect to the cost of fermentation, w_1/b_1, where the protein costs are weighted by the inverse yields of the pathways. Since w_C increases with decreasing nutrient levels, the sign of $Q - w_C$ might change when one shifts from good carbon sources to poor ones. For the realistic case where $Q > 0$, in specific, the optimal solution shifts from fermentation to respiration as w_C increases, i.e. as carbon is limited.

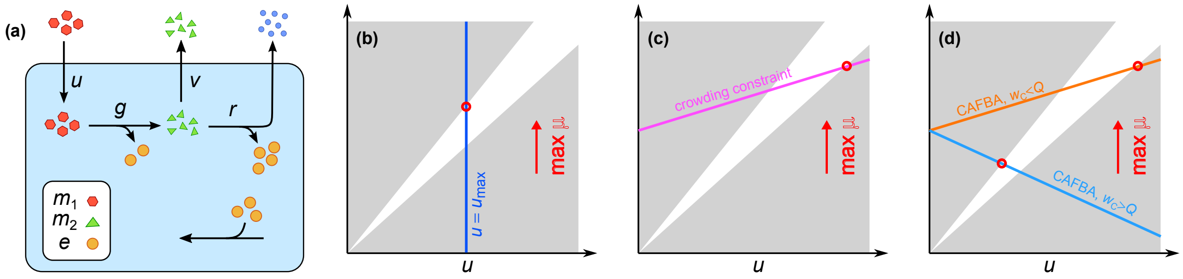

FIG. N2. **(a)** Coarse grained model for the overflow metabolism. A metabolite m_1 can be consumed to provide "energy" e plus other metabolic products m_2. These other products can be either further processed to yield more energy e (plus waste), or excreted. The metabolites e are then used by the cell to grow. **(b)-(d)** Solution space of the model imposing the steady state constraints $\mathrm{d}[m_1]/\mathrm{d}t = \mathrm{d}[m_2]/\mathrm{d}t = \mathrm{d}[e]/\mathrm{d}t = 0$. Grey areas denote infeasible regions. Feasible solution (white area) are delimited by the linear bounds $b_1 u \leq \mu \leq (b_1 + ab_2)u$, see Eq. (31). As μ does not have an upper limit, an extra constraint is required in order to maximize the growth rate μ. We consider 3 different scenarios. **(b)** Maximization of μ subject to a maximum intake flux (standard FBA). **(c)** Maximization of μ subject to a molecular crowding constraint; the slope of the purple line is always positive. **(d)** Maximization of μ subject to a proteome allocation constraint, with the lines corresponding to the cases $w_C < Q$ and $w_C > Q$ shown explicitly. Optimal solutions for each of the examples displayed are shown as red circles.

SUPPLEMENTARY FIGURES

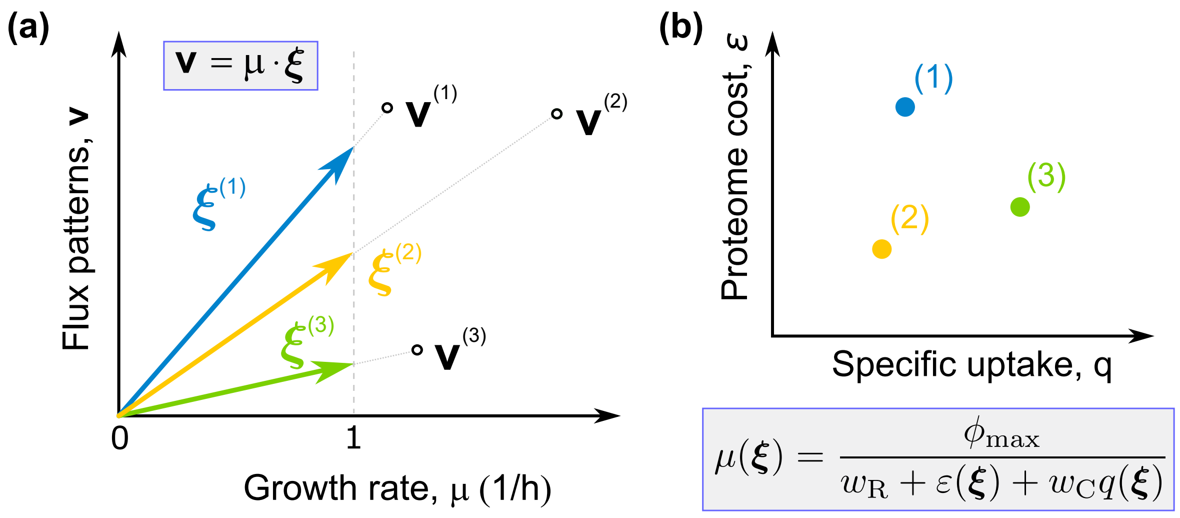

FIG. S1. **Metabolic states and specific costs. (a)** Any flux pattern $\mathbf{v}$ with a growth rate $\mu > 0$ (black circles) can be decomposed as the product of the growth rate and a "metabolic state" vector $\boldsymbol{\xi}$ (colored arrows), so that $\mathbf{v} = \mu \cdot \boldsymbol{\xi}$. The entries of $\boldsymbol{\xi}$ represent the total reaction activities (mmol of products produced) per unit of cellular dry mass. **(b)** For each metabolic state $\boldsymbol{\xi}$ (such as the three states shown in panel (a)) one can compute the specific carbon uptake rate $q(\boldsymbol{\xi}) = \xi_{\mathrm{C}}$ and the specific protein cost $\varepsilon = \sum_{i \in E} w_i |\xi_i|$. The growth rate μ corresponding to the metabolic state $\boldsymbol{\xi}$ is then computed from q and ϵ through Eq. (4) from the Main Text, and with it the corresponding flux vector $\mathbf{v} = \mu \cdot \boldsymbol{\xi}$.

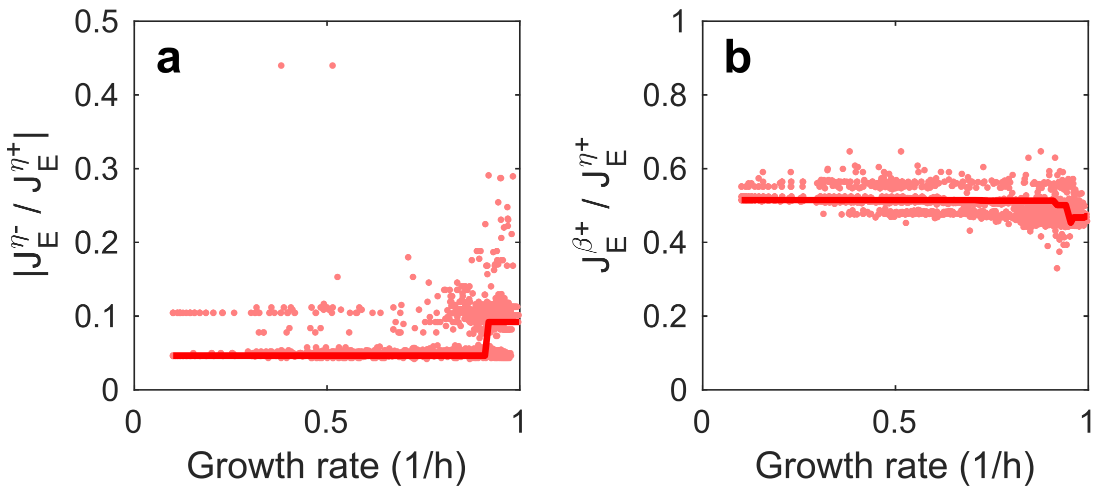

FIG. S2. **Energy production partitioning.** The flux decomposition into energy- and biomass-associated vectors (Fig. 3a) is only useful if J_E either dominates the total energy budget of the cell (e.g. the biosynthetic cost of the biomass components is small with respect to J_E) or if it represents a fixed fraction of the total energy budget. We define the vector $\mathbf{S}_{ATP}$, whose components $S_{ATP,i}$ represent the stoichiometric coefficient of ATP in reaction i. (We exclude the biomass reaction and the ATP maintenance reaction.) For a given flux v_i, $S_{ATP,i}v_i$ indicates the ATP production (if positive) or consumption (if negative) flux. When summing over all reactions, we can separate the positive and negative contributions as $\mathbf{S}_{ATP} \cdot \mathbf{v} = \sum_i S_{ATP,i}v_i = (\mathbf{S}_{ATP} \cdot \mathbf{v})^+ + (\mathbf{S}_{ATP} \cdot \mathbf{v})^-$, with $(\mathbf{S}_{ATP}\mathbf{v})^+ = \sum_i S_{ATP,i}v_i\Theta(S_{ATP,i}v_i)$, $(\mathbf{S}_{ATP} \cdot \mathbf{v})^- = \sum_i S_{ATP,i}v_i\Theta(-S_{ATP,i}v_i)$ and with $\Theta(x)$ denoting the Heaviside theta function. Hence, $(\mathbf{S}_{ATP} \cdot \mathbf{v})^+$ measures the total ATP synthesized by the flux vector $\mathbf{v}$. Since each flux vector can be decomposed as $\mathbf{v} = \boldsymbol{\eta} \cdot J_E + \boldsymbol{\beta} \cdot \mu$, the total ATP production J_E can be expressed as $J_E = \mathbf{S}_{ATP}\mathbf{v} = (\mathbf{S}_{ATP}\boldsymbol{\eta}) \cdot J_E + (\mathbf{S}_{ATP}\boldsymbol{\beta}) \cdot \mu$. By further decomposing the scalar products into positive and negative parts we obtain four terms, namely $J_E^{\boldsymbol{\eta}\pm} = (\mathbf{S}_{ATP} \cdot \boldsymbol{\eta})^{\pm} \cdot J_E$ and $J_E^{\boldsymbol{\beta}\pm} = (\mathbf{S}_{ATP} \cdot \boldsymbol{\beta})^{\pm} \cdot \mu$, with the two constraints $J_E^{\boldsymbol{\eta}+} + J_E^{\boldsymbol{\eta}-} = J_E$ and $J_E^{\boldsymbol{\beta}+} + J_E^{\boldsymbol{\beta}-} = 0$.

(a) $|J_E^{\boldsymbol{\eta}-}/J_E^{\boldsymbol{\eta}+}|$ is below 0.1 for most of the samples, implying that $J_E^{\boldsymbol{\eta}+} \approx J_E$. **(b)** $J_E^{\boldsymbol{\beta}+}/J_E^{\boldsymbol{\eta}+} \approx 1/2$ for all metabolic states sampled in Fig. 3 (Main Text). This means that about 50% of the ATP generated in the cell is used to fuel the biosynthetic reactions included in the vector $\boldsymbol{\beta}$, independently of the growth rate. In turn, considering the constraints mentioned above, one concludes that, in terms of absolute fluxes, about 1/3 (resp. 2/3) of the in-taken carbon goes through pathways that contribute to the overall production of energy (resp. biomass).

SUPPLEMENTARY TABLES

Symbol	Description
μ	Growth rate (units: per hour).
J_C	Carbon (lactose) intake flux, in units of mmol lactose per dry weight per hour.
ϕ_C	Mass fraction of catabolic proteins, responsible for uptaking the carbon source with a flux J_C.
w_C	Protein cost associated to carbon intake. Its value varies in growth media with different carbon source availability: a larger value indicates lower carbon concentrations and/or quality.
ϕ_{NC}	Fraction of non-limited catabolic proteome ($\phi_{NC} = 1 - \phi_C$). In Eq. (2) it is assumed to depend linearly on growth rate as $\phi_{NC} = \phi_{NC,0} + \epsilon_{NC}\mu$.
v_i	Flux of each metabolic reaction (labeled by the index i) included in the genome-scale model of metabolism studied in this work (iJR904). This usually excludes carbon uptake (whose flux is J_C).
$\mathbf{v}$	Vector whose entries are all metabolic fluxes v_i.
w_i	Protein cost for individual enzymatic reactions (the proteome E-sector, as described in the text). Under the "uniform" or "homogeneous weights" approximation, they are all taken to be the same, $w_i = w_E = 8.3 \cdot 10^{-4} \mathrm{g_{DW}}$h/mmol.
w_R	Protein cost associated to ribosome biosynthesis. (We use $w_R = 0.169/h$ in all simulations.)
$\phi_{\max}$	Cap on the fraction of proteins that can be allocated in a condition-dependent manner. ($\phi_{\max} = 0.484$ in all simulations.)
q	Specific carbon flux, $q = J_C/\mu$. It specifies the amount of carbon (in our case lactose) intaken per unit of dry mass, and has units of mmol lactose per gram of dry weight. In other words, the production of 1 gram of dry cells requires q mmol of lactose. It is also inversely proportional to the growth yield, usually defined as grams of dry weight per gram of substrate.
ε	Protein cost of the enzymatic reaction, $\varepsilon = \sum_{i \in E} w_i \vert v_i \vert / \mu$.
$\mathcal{C}$	Total protein cost, including the one associated to carbon uptake (qw_C) and the metabolic one (ε).
$\boldsymbol{\xi}$	Metabolic state of the cell, $\boldsymbol{\xi} = \mathbf{v}/\mu$. Each component ξ_i has unit of mmol per gram of dry weight, and specifies the extent of reaction i (mmol of molecules transformed) per gram of biomass generated.
J_E	Energy production flux (ATP hydrolysis rate). Units: mmol ATP per grams of dry weight per hour.
σ	Growth-associated ATP hydrolysis rate (units: mmol ATP hydrolyzed per gram of dry weight). It relates the energy production flux J_E to growth rate, $J_E = \sigma\mu$.
$J_{C\to E}$	Carbon flux associated to energy production.
$J_{C\to B}$	Carbon flux associated to the production of biomass precursors. This includes the production of energy necessary to fuel the biosynthetic pathways.
q_E	Energy-associated specific carbon intake, $q_E = J_{C\to E}/J_E = J_{C\to E}/(\sigma\mu)$ (mmol of lactose per mmol of ATP synthesized).
q_B	Biomass-associated specific carbon intake, $q_B = J_{C\to B}/\mu$ (mmol lactose per gram of biomass precursors)
$\boldsymbol{\eta}, \boldsymbol{\beta}$	Energy- and biomass-associated vectors ($\boldsymbol{\eta}$ and $\boldsymbol{\beta}$, respectively) describing the metabolic state of the cell when the fluxes to energy and biomass precursors are considered separately.

TABLE S1. List of symbols and parameters used in the Main Text.

[1] Mori M, Ponce-de-Leon M, Pereto J, Montero F (2016) Metabolic complementation in bacterial communities: necessary conditions and optimality. Frontiers Microbiol 7:1553.

[2] Mori M, Hwa T, Martin OC, De Martino A, Marinari E (2016) Constrained allocation flux balance analysis. PLoS Comp Biol 12:e1004913.

[3] Beg QK, Vazquez A, Ernst J, de Menezes MA, Bar-Joseph Z, Barabasi AL, Oltvai ZN (2007) Intracellular crowding defines the mode and sequence of substrate uptake by Escherichia coli and constrains its metabolic activity. Proc Natl Acad Sci USA 104:12663-8.

[4] Vazquez A, Beg QK, Ernst J, Bar-Joseph Z, Barabasi AL, Boros LG, Oltvai ZN (2008) Impact of the solvent capacity constraint on E. coli metabolism. BMC Sys Biol 2:7.

[5] Woldringh CL, Binnerts JS, Mans A (1981) Variation in Escherichia coli buoyant density measured in Percoll gradients. J Bacteriol 148:58-63.

[6] Basan M, Zhu M, Dai X, Warren M, Sevin D, Wang YP, Hwa T (2015) Inflating bacterial cells by increased protein synthesis. Mol Sys Biol 11:836.
